# Performance of ChatGPT, Bard, Claude, and Bing on the Peruvian National Licensing Medical Examination: a cross-sectional study

**DOI:** 10.3352/jeehp.2023.20.30

**Published:** 2023-11-20

**Authors:** Betzy Clariza Torres-Zegarra, Wagner Rios-Garcia, Alvaro Micael Ñaña-Cordova, Karen Fatima Arteaga-Cisneros, Xiomara Cristina Benavente Chalco, Marina Atena Bustamante Ordoñez, Carlos Jesus Gutierrez Rios, Carlos Alberto Ramos Godoy, Kristell Luisa Teresa Panta Quezada, Jesus Daniel Gutierrez-Arratia, Javier Alejandro Flores-Cohaila

**Affiliations:** 1 Escuela de Medicina, Universidad Cientifica del Sur, Lima, Peru; 2 Sociedad Científica de Estudiantes de Medicina de Ica, Universidad Nacional San Luis Gonzaga, Ica, Peru; 3 Universidad Nacional de Cajamarca, Cajamarca, Peru; 4 Academic Department, USAMEDIC, Lima, Peru; 5 Neurogenetics Research Center, Instituto Nacional de Ciencias Neurologicas, Lima, Peru; Hallym University, Korea

**Keywords:** Medical education, Educational measurement, Artificial intelligence, Peru

## Abstract

**Purpose:**

We aimed to describe the performance and evaluate the educational value of justifications provided by artificial intelligence chatbots, including GPT-3.5, GPT-4, Bard, Claude, and Bing, on the Peruvian National Medical Licensing Examination (P-NLME).

**Methods:**

This was a cross-sectional analytical study. On July 25, 2023, each multiple-choice question (MCQ) from the P-NLME was entered into each chatbot (GPT-3, GPT-4, Bing, Bard, and Claude) 3 times. Then, 4 medical educators categorized the MCQs in terms of medical area, item type, and whether the MCQ required Peru-specific knowledge. They assessed the educational value of the justifications from the 2 top performers (GPT-4 and Bing).

**Results:**

GPT-4 scored 86.7% and Bing scored 82.2%, followed by Bard and Claude, and the historical performance of Peruvian examinees was 55%. Among the factors associated with correct answers, only MCQs that required Peru-specific knowledge had lower odds (odds ratio, 0.23; 95% confidence interval, 0.09–0.61), whereas the remaining factors showed no associations. In assessing the educational value of justifications provided by GPT-4 and Bing, neither showed any significant differences in certainty, usefulness, or potential use in the classroom.

**Conclusion:**

Among chatbots, GPT-4 and Bing were the top performers, with Bing performing better at Peru-specific MCQs. Moreover, the educational value of justifications provided by the GPT-4 and Bing could be deemed appropriate. However, it is essential to start addressing the educational value of these chatbots, rather than merely their performance on examinations.

## Graphical abstract

[Fig f2-jeehp-20-30]

## Introduction

### Background/rationale:

Recently, there has been growing interest in the performance of chatbots such as ChatGPT, Bing, Bard, and Claude on national licensing medical examinations (NLMEs). Some studies have reported outstanding performance in which chatbots matched and even outperformed medical examinees [1-3]. However, there is a lack of studies comparing the performance of different chatbots, which hinders their potential use in classifying examination complexity [4]. Furthermore, studies exploring the quality of chatbot justifications in multiple-choice questions (MCQs), focusing on their educational value, are lacking in the current literature. In this study, we aimed to address these issues.

### Objectives

In this study, we aimed to describe the performance and evaluate the educational value of justifications provided by ChatGPT (using GPT-3 and GPT-4), Bard, Claude, and Bing on the Peruvian National Licensing Examination (P-NLME) of 2023. The following objectives were addressed to describe the performance of chatbots on the P-NLME under 3 attempts for each chatbot: to identify factors associated with correct answers provided by chatbots in the P-NLME; and to assess the educational value of the justifications provided by the 2 top-performing chatbots in terms of certainty, usefulness, and potential use in the classroom setting.

## Methods

### Ethics statement

We did not seek approval from an institutional review board or informed consent for this study because we analyzed the performance of chatbots on an NMLE rather than conducting human-subject research.

### Study design

This cross-sectional analytical study compared the accuracy of chatbots (GPT-3 and GPT-4, Bing, Claude, and Bard) with the historical performance of examinees on an NMLE, which has been previously published [5]. Additionally, based on previous research, we assessed the factors associated with correct answers [1] and evaluated the educational value of the justifications provided by chatbots [6].

### Setting and procedures

On July 25, 2023, we entered the 2023 P-NLME into the selected chatbots (GPT-3.5 and GPT-4, Bing, Claude, and Bard) 3 times, as the responses provided by chatbots were not deterministic. The answers provided by chatbots were saved in Microsoft Excel (Microsoft Corp.), and at least 2 medical educators categorized the MCQs according to the area and type of MCQ and if they required Peru-specific knowledge. Subsequently, the justifications provided by the 2 top-performing chatbots (Bing and GPT-4) were analyzed using items from previously published instruments that assess the quality of open-access medical education resources [7]. The prompt used is availableat Supplement 1. One hundred- eight MCQ items are available at Supplement 2.

### Participants

The chatbots were counted as participants, resulting in 15 participants (3 attempts per chatbot). The evaluator team comprised 4 medical educators with training in developing and evaluating MCQs.

### Variables

The dependent variables were the answers provided by the chatbot (correct or incorrect), defined as the choice selected by at least 3 medical educators. The independent variables were the area of the MCQ, the type of item, whether Peru-specific knowledge was required, and the educational value of the justifications.

### Data sources/measurements

MCQs from the P-NLME were analyzed for the area of medicine to which the MCQs belonged, including surgery, internal medicine, pediatrics, obstetrics and gynecology, public health, and emergency medicine, according to the P-NLME specifications table [8]. For the item type, we categorized MCQs into 2 categories: those that solely assessed the recall of information and those that evaluated the application of knowledge involving decision-making in the form of diagnosis or treatment [9]. Regarding the requirement for Peru-specific knowledge, MCQs were categorized as “yes” if the MCQs assessed or required knowledge specific to Peru, such as epidemiological, clinical practice guidelines, or diseases restricted to this country. Finally, to evaluate the educational value, we adopted the Academic Life in Emergency Medicine (ALiEM) using the Approved Instructional Resources (AIR) [7,10]. We considered educational value as the certainty, usefulness, and potential use of the responses provided by chatbots. Certainty was defined as the accuracy of the information provided in each chatbot response (GPT-4 and Bing). Usefulness was defined as the number of educational pearls (stand-alone clinical relevant details) and potential use in the classroom, referring to the potential to use the response in hypothetical classes. The categorization of MCQs and assessment of educational value were carried out by at least 2 independent authors (J.A.F.C., C.J.G.R., C.A.R.G., K.T.P.Q., J.D.G.A.), who had previously experienced training medical examinees for the ENAM (Examen Nacional de Medicina). The rating scale employed to assess educational value is available in Supplement 3.

### Study size

We analyzed all 180 MCQs from the P-NLME of 2023. Therefore, a sample size calculation was not required.

### Statistical methods

Descriptive statistics were used to analyze the scores for each chatbot and the rest of the categories. They are presented as absolute values along with their frequencies. We conducted an agreement test for each chatbot using the Fleiss kappa. We considered a kappa <0.20 as indicating no agreement, 0.21 to 0.39 as minimal, 0.40 to 0.59 as weak, 0.60 to 0.79 as moderate, 0.80 to 0.90 as strong, and above 0.90 as almost perfect agreement [11]. Then, inferential statistics were employed, using the chi-square test to compare the highest rating on certainty, usefulness, and potential use between Bing and GPT-4, considering a P-value ≤0.05 as statistically significant. Additionally, we employed a bivariate logistic regression model to identify potential factors associated with correct answers for each chatbot’s best attempt. All analyses were conducted using RStudio ver. 4.1.2 (RStudio) (Supplement 4).

## Results

### Performance and agreement between chatbots

**Fig. 1.**
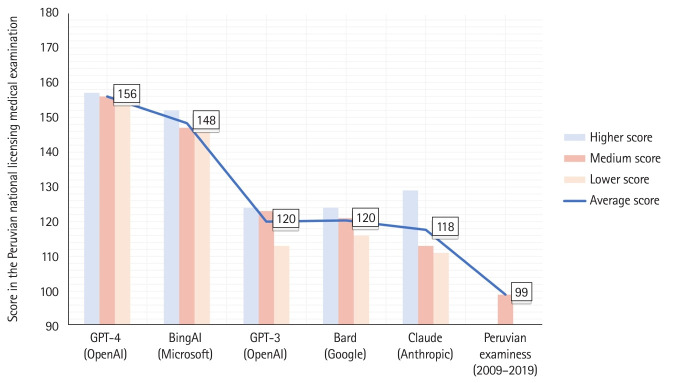
Scores obtained in the Peruvian national licensing medical examination by chatbots, compared with the average score of Peruvian examinees from 2009 to 2019.

As shown in Fig. 1, GPT-4 and Bing had the highest average scores (156 [86.7%] and 148 [82.2%] out of 180, respectively). For other chatbots, such as GPT-3, Bard, and Claude, the average score ranged from 118 (65.6%) to 120 (66.7%). Regarding scores on each of the 3 attempts, they were as follows: Bard: 113, 119, and 122; Claude: 109, 111, and 127; GPT-3: 112, 122, and 123; Bing: 144, 145, and 150; and GPT-4: 153, 154, and 155. In contrast, the average score for Peruvian examinees from 2009 to 2019 was 99 (55%) [5].

The level of agreement among various chatbots is displayed in Table 1. Most chatbots exhibited substantial agreement, except for Bard, which showed only moderate agreement. When analyzing the remaining categories, the level of agreement ranged from moderate to substantial for all chatbots. In emergency medicine, GPT-4 demonstrated almost perfect agreement, whereas Bing and Claude showed no agreement.

Table 2 shows the best performance for each chatbot in the various categories. GPT-4 outperformed other chatbots in all categories except obstetrics and gynecology, and public health. These exceptions occurred in instances requiring Peru-specific knowledge or for questions that evaluated recall rather than applying knowledge. In these specific cases, Bing outperformed GPT-4.

### Factors associated with correct answers

Table 3 presents bivariate regression models for each chatbot. Although we analyzed multiple categories, some noteworthy associations emerged. Specifically, questions solved by GPT-4 that required Peru-specific knowledge had lower odds (odds ratio [OR], 0.23; 95% confidence interval [CI], 0.09–0.61) of being correct. Similarly, for questions solved by Bard that required the application of knowledge, the odds of being correct were lower (OR, 0.43; 95% CI, 0.16–0.99).

### The educational value of responses provided by GPT-4 and Bing

We selected the best attempts from GPT-4 and Bing, including their corresponding responses and justifications, to assess their educational value. The findings are summarized in Table 4. Medical educators considered Bing’s justifications superior to “full of educational pearls” compared to GPT-4 (42 versus 59). However, GPT-4 outperformed Bing regarding the number of responses containing 3 or more educational pearls (86 versus 59). Therefore, although the 2 chatbots exhibited different strengths when analyzed by these categories (summing up “full of educational pearls” and “3 or more educational pearls”), no statistically significant difference was observed when the metrics were combined (χ^2^=1.284, P=0.257). Furthermore, in the item “Potential use of the justification provided by chatbots in classes,” fewer than 20% of the justifications were considered as “I would not use anything” (13.33% for GPT-4 and 12.22% for Bing). For “Yes, I would use the entire explanation,” there were no significant differences between GPT-4 and Bing (χ^2^=1.284, P=0.112).

All research data are available at Dataset 1.

## Discussion

### Key results

Our major findings are as follows: (1) we found that the chatbots’ average performance was above the historical performance of Peruvian examinees in the P-NLME, with GPT-4 and Bing being the top performers; (2) we did not detect any associations between the correct answers and the specific areas of the MCQs, item type, or whether they required Peru-specific knowledge for the majority of chatbots, except GPT-4; and (3) there were no statistically significant differences in terms of certainty between GPT-4 and Bing (P=0.777), nor in the potential use of responses in the classroom (P=0.112). Furthermore, there were no differences regarding the presence of ≥3 educational pearls per GPT-4 and Bing response. These findings suggest the superiority of GPT-4 and Bing in written assessments in medical education regarding performance and educational value.

### Interpretation

The outstanding performance of chatbots, mainly GPT-4 (87.2%) and Bing (84.4%), is not surprising, as previous studies have reported similar outcomes [1,2]. We hypothesized that internet access may impact specific categories, as it is well known that the performance of chatbots depends on the dataset used to train them and because Bing has access to the internet. This is supported by the fact that MCQs that required Peru-specific knowledge were associated with lower odds of correct answers provided by GPT-4, whereas this tendency was not observed in Bing. This suggests that for educational purposes, commercial chatbots may need to be trained or tailored to a specific setting, such as epidemiological data or beliefs particular to a country.

Regarding our major topic of interest, we evaluated the educational value of justifications provided by GPT-4 and Bing. We found that the outstanding performance was not solely quantitative but also qualitative, with chatbots providing “educational pearls” in more than 50% of their justifications. Furthermore, the educators considered using the justifications in their classes. This shows the feasibility of their use in the teaching and learning process for medical education, a field not yet explored in the literature but suggested in a previous report [12].

### Comparison with previous studies

Previous studies have compared the performance of commercial chatbots on NLMEs, with similar findings for GPT-4. On the Japanese NLME, it scored 79.9%, while examinees scored 84.9% [13]; on the Chinese NLME, it scored 84% [3], and it scored 83.46% and 84.75% on the United States Medical Licensing Examination Step 1 and Step 2, respectively [2]. Furthermore, a previous study showed that GPT-4 scored 86% on the Peruvian NLME, whereas examinees scored 54% [1]. Therefore, the performance of GPT-4 across several NLMEs appears to be homogeneous and independent of the setting or language. Regarding educational value, one study showed that the acceptability of justifications provided by GPT-3.5 was 52% [6]. No other studies have assessed this; therefore, a comprehensive comparison is lacking.

### Limitations

While our study aimed to compare chatbots with examinees’ historical performance on the P-NLME, this approach could introduce selection bias. The findings may not be interpretable as we compared performances from different years. However, it is worth noting that the P-NLME is designed based on a standardized test blueprint, intending to measure the same constructs consistently across years, reducing the potential bias. Moreover, the assessment of “educational value” was conducted by medical educators, a factor that may introduce evaluation bias due to subjective interpretations. To mitigate this bias, the evaluation process was conducted in duplicate.

### Generalizability

Given prior research, it is plausible that our findings may be extended to other NLMEs that adhere to a meticulous development process, such as those from Peru, China, Japan, and the United States. Nevertheless, it is pivotal to note that our educational value findings involved trained medical educators experienced in crafting and assessing MCQs, potentially limiting generalizability across all contexts. Additionally, considering the rapid advancement of chatbots, their capabilities might supersede those of previous iterations within a few weeks or months after this study’s publication.

### Implications and suggestions

We employed and compared all available commercial chatbots; thus, we offer some perspectives on how each chatbot performs in questions regarding medical knowledge. This can inform educators and students about which chatbots are more suitable for academic tasks. Additionally, we provided evidence that GPT-4 had lower odds of obtaining correct answers when they required Peru-specific knowledge, a phenomenon not observed in Bing. Therefore, this may suggest that Bing may be more suitable for non-English medical education tasks, such as explaining topics, developing MCQs, or other endeavors not yet explored. Future research should address this in more specific tasks, such as decision-making related to country-specific guidelines. We found that the justifications offered by both the GPT-4 and Bing were deemed valuable by medical educators. However, it is important to recognize that our conclusions may not be universally applicable, as our own inherent biases and paradigms influenced them, and only 2 authors assessed each justification. Consequently, future research should explore the educational value of chatbot justifications by gathering perspectives from a broader spectrum of educators, ranging from novices to experienced educators.

### Conclusion

Among the chatbots, GPT-4 and Bing were the top performers, with Bing performing better at Peru-specific MCQs. Moreover, the educational value of justifications provided by the GPT-4 and Bing could be deemed appropriate. However, it is essential to start addressing the educational value of these chatbots, rather than merely their performance on examinations.

## Figures and Tables

**Figure f2-jeehp-20-30:**
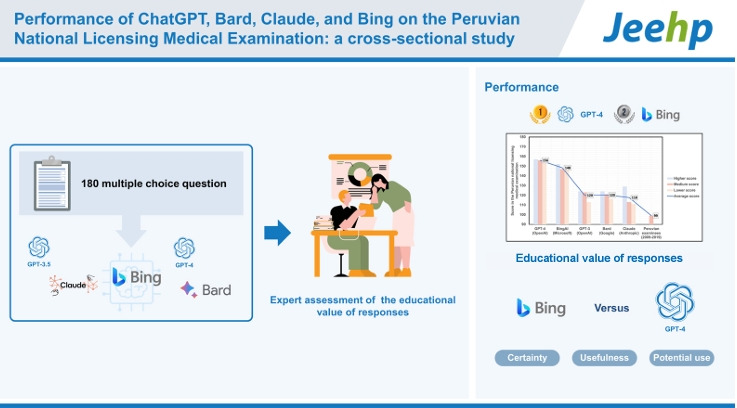


**Table 1.** Agreement between the 3 attempts of each chatbot calculated using the Fleiss kappa

	GPT-4	Bing	GPT-3	Claude	Bard
Total	0.647	0.668	0.700	0.714	0.574
Areas					
Surgery	0.100	0.655	0.769	0.843	0.688
Internal medicine	0.638	0.837	0.669	0.678	0.632
Pediatrics	0.571	0.595	0.550	0.847	0.417
Obstetrics & gynecology	0.745	0.396	0.733	0.699	0.697
Public health	0.709	0.844	0.699	0.741	0.096
Emergency medicine	1.000	0.111	0.832	-0.007	0.495
Type of item					
Recall	0.533	0.782	0.665	0.623	0.321
Application of knowledge	0.688	0.632	0.708	0.735	0.628

**Table 2.** Total and subgroup scores of the best attempt of each chatbot

	GPT-4	Bing	Claude	Bard	GPT-3	Total
Total	157 (87.2)	152 (84.4)	129 (71.6)	124 (68.8)	124 (68.8)	180
Area						
Surgery	26 (96.2)	22 (81.48)	17 (62.9)	20 (74.07)	19 (70.3)	27
Internal medicine	68 (90)	68 (90)	59 (78.6)	52 (69.3)	55 (73.3)	75
Pediatrics	14 (77.7)	11 (61.1)	11 (61.1)	11 (61.1)	10 (55.5)	18
Obstetrics & gynecology	24 (68.5)	27 (77.1)	17 (48.5)	17 (48.5)	21 (60)	35
Public health	18 (85.7)	16 (76)	18 (85.7)	17 (80.9)	15 (71.4)	21
Emergency medicine	7 (87.5)	8 (100)	7 (87.5)	7 (87.5)	4 (50)	8
Peruvian knowledge						
Required	24 (70.5)	27 (79.4)	24 (70.5)	21 (61.7)	21 (61.7)	34
Not required	133 (91.09)	125 (85.6)	105 (71.9)	103 (70.5)	103 (70.5)	146
Type of item						
Recall	30 (78.9)	32 (84.2)	30 (78.9)	31 (81.5)	27 (71.05)	38
Application of knowledge	127 (89.4)	120 (84.5)	99 (69.7)	94 (66.1)	97 (68.3)	142

Values are presented as number (%) or number.

**Table 3.** Factors associated with correct answers provided by chatbots in a bivariate logistic regression model

	GPT-4	Bing	Claude	Bard	GPT-3
Area					
Surgery	Ref	Ref	Ref	Ref	Ref
Internal medicine	0.37 (0.02 to 2.25)	2.21 (0.60 to 7.63)	2.17 (0.82 to 5.64)	0.79 (0.28 to 2.07)	1.16 (0.42 to 3.00)
Pediatrics	0.13 (0.01 to 1.02)	0.36 (0.09 to 1.37)	1.08 (0.27 to 3.23)	1.82 (0.15 to 1.99)	0.53 (0.15 to 1.83)
Obstetrics & gynecology	0.13 (0.01 to 0.82)	1.53 (0.36 to 6.86)	0.71 (0.24 to 2.04)	0.42 (0.13 to 1.27)	0.88 (0.28 to 2.70)
Public health	0.23 (0.11 to 1.96)	0.72 (0.17 to 3.02)	3.53 (0.90 to 17.79)	1.49 (0.38 to 6.50)	1.05 (0.30 to 3.83)
Emergency medicine	0.27 (0.01 to 7.38)	Not estimable	4.12 (0.60 to 82.89)	2.45 (0.34 to 50.03)	0.42 (0.08 to 2.17)
Peruvian knowledge					
Not required	Ref	Ref	Ref	Ref	Ref
Required	0.23 (0.09 to 0.61)^a)^	0.65 (0.26 to 1.78)	0.94 (0.42 to 2.21)	0.67 (0.31 to 1.50)	0.67 (0.31 to 1.50)
Type of item					
Recall	Ref	Ref	Ref	Ref	Ref
Application of knowledge	2.25 (0.84 to 5.71)	1.02 (0.35 to 2.60)	0.61 (0.25 to 1.39)	0.43 (0.16 to 0.99)	0.88 (0.39 to 1.89)

Values are presented as odds ratio (95% confidence interval). Ref, reference.

a) The odds ratio was statistically significant.

**Table 4.** Ratings of certainty, usefulness, and potential use in class for the best GPT-4 and Bing scores by 2 medical educators

	GPT-4	Bing	P-value
Item 1: Certainty of the justification provided by chatbots			
This is not the correct answer, and the information is wrong.	7 (3.89)	7 (3.89)	-
Not the right answer, but the information is somewhat correct.	16 (8.89)	21 (11.67)	-
This is the correct answer, but the information is wrong.	6 (3.33)	3 (1.67)	-
It is the correct answer, and the information is accurate.	151 (83.89)	149 (82.78)	0.777
Item 2: Usefulness of the justification provided by chatbots			
It has no educational pearls.	5 (2.78)	9 (5.00)	-
There are about 1–2 educational pearls or important concepts that a competent physician should know.	47 (26.11)	53 (29.44)	-
There are quite a few (more than 3) educational pearls that a competent physician should know.	86 (47.78)	59 (32.78)	0.037^a)^
The entire contents are educational pearls that a competent physician should know.	42 (23.33)	59 (32.78)	0.046^a)^
Item 3: Potential use of the justification provided by chatbots in classes			
No, I wouldn’t use anything.	24 (13.33)	22 (12.22)	-
I would use some of this as a guide.	82 (45.56)	69 (38.33)	-
Yes, I would use the entire explanation.	74 (41.11)	89 (49.44)	0.112

Values are presented as number (%). P-value for the chi-square test comparing GPT-4 and Bing on the highest rating of each item.

a) The difference is statistically significant according to the chi-square test.

## Footnotes

**Data availability**
Data files are available from Harvard Dataverse: https://doi.org/10.7910/DVN/RVDNLX Dataset 1. The data file contained all available information for the analysis.

## Supplementary materials

Supplementary files are available from Harvard Dataverse: https://doi.org/10.7910/DVN/RVDNLX

Supplement 1. Prompts for all chatbots.

Supplement 2. Multiple-choice question dataset.

Supplement 3. Instruments and rating scales used to assess the multiple-choice questions.

Supplement 4. RStudio script for the analysis.

Supplement 5. Audio recording of the abstract.

## References

[1] Flores-Cohaila JA, Garcia-Vicente A, Vizcarra-Jimenez SF, De la Cruz-Galan JP, Gutierrez-Arratia JD, Quiroga Torres BG, Taype-Rondan A (2023). Performance of ChatGPT on the Peruvian National Licensing Medical Examination: cross-sectional study. JMIR Med Educ.

[2] Nori H, King N, McKinney SM, Carignan D, Horvitz E (2023). Capabilities of GPT-4 on medical challenge problems. arXiv [Preprint].

[3] Wang H, Wu W, Dou Z, He L, Yang L (2023). Performance and exploration of ChatGPT in medical examination, records and education in Chinese: pave the way for medical AI. Int J Med Inform.

[4] Kumari A, Kumari A, Singh A, Singh SK, Juhi A, Dhanvijay AK, Pinjar MJ, Mondal H (2023). Large language models in hematology case solving: a comparative study of ChatGPT-3.5, Google Bard, and Microsoft Bing. Cureus.

[5] Mendoza-Chuctaya G, Calla-Torres M, Ramos KR, Mejia CR (2021). [Examen Nacional de Medicina (ENAM): analysis of the last decade of theoretical evaluations in future doctors in Peru]. Acta Med Peru.

[6] Huh S (2023). Are ChatGPT’s knowledge and interpretation ability comparable to those of medical students in Korea for taking a parasitology examination?: a descriptive study. J Educ Eval Health Prof.

[7] Ediger D, Sumpter R, Bridwell RE, Belcher CN (2020). Academic Life in Emergency Medicine (ALiEM) Blog and Podcast Watch: infectious diseases. Cureus.

[8] Asociacion Peruana de Facultades de Medicina (2020). ENAM specifications table [Internet]. https://www.aspefam.org.pe/enam/enam2023ord/tabla_enam_03.12.2023.pdf.

[9] Lai H, Gierl MJ, Touchie C, Pugh D, Boulais AP, De Champlain A (2016). Using automatic item generation to improve the quality of MCQ distractors. Teach Learn Med.

[10] Chan TM, Grock A, Paddock M, Kulasegaram K, Yarris LM, Lin M (2016). Examining reliability and validity of an online score (ALiEM AIR) for rating free open access medical education resources. Ann Emerg Med.

[11] McHugh ML (2012). Interrater reliability: the kappa statistic. Biochem Med (Zagreb).

[12] Lee H (2023). The rise of ChatGPT: Exploring its potential in medical education. Anat Sci Educ.

[13] Takagi S, Watari T, Erabi A, Sakaguchi K (2023). Performance of GPT-3.5 and GPT-4 on the Japanese Medical Licensing Examination: comparison study. JMIR Med Educ.

