# Supplementary material for: Performance of ChatGPT, Bard, Claude, and Bing on the Peruvian National Licensing Medical Examination: a cross-sectional study
Source: J Educ Eval Health Prof. 2023 Nov 20;20:30. doi: 10.3352/jeehp.2023.20.30 (PMC11009012; doi:10.3352/jeehp.2023.20.30)
Supplement: Supplementary file 2 — Supplement 1. Prompts for all chatbots. [file jeehp-20-30-suppl1.docx]

# Supplementary material 1: Prompt used in chatbots.

Prompt in Spanish (Original):

{ 1. Dime quien eres: BingAI / GPT-4 o GPT-3.5 / ClaudeAI / Google Bard

2. Esta es una pregunta de tipo test de opcion multiple

3. La resolveremos, para hacer esto seguiremos el siguiente proceso: - Identificaremos el objetivo de aprendizaje - Identifica la alternativa más correcta

4. En base de esto da un comentario que tenga la estructura: - Hola Soy [Quien eres]. El objetivo de la pregunta era [Objetivo]. La clave correcta aqui era [Clave correcta] [Sustento de porque es]. Las otras eran incorrectas por [Clave + Sustento de porque fueron incorrectas] - [Clave Correcta] }

Prompt translated to English

{ 1. Tell me who you are: BingAI / GPT-4 or GPT-3.5 / ClaudeAI / Google Bard

2. This is a multiple-choice quiz question

3. We will solve it, to do this we will follow the following process: - We will identify the learning objective - Identify the most correct alternative

4. Based on this give a commentary that has the structure: - Hello I am [Who you are]. The objective of the question was [Objective]. The correct key here was [Correct key] [Substantiation of why it is]. The others were incorrect because of [Key + Substantiation of why they were incorrect] - [Correct Key] }
