# Supplementary material for: Performance of ChatGPT, Bard, Claude, and Bing on the Peruvian National Licensing Medical Examination: a cross-sectional study
Source: J Educ Eval Health Prof. 2023 Nov 20;20:30. doi: 10.3352/jeehp.2023.20.30 (PMC11009012; doi:10.3352/jeehp.2023.20.30)
Supplement: Supplementary file 4 — Supplement 3. Instruments and rating scales used to assess the multiple-choice questions. [file jeehp-20-30-suppl3.docx]

# Supplement 3. Rating scale for justifications provided by chatbots

|  | 1 | 2 | 3 | 4 |
| --- | --- | --- | --- | --- |
| Certainty | This is not the correct answer, and the information is wrong. | Not the right answer, but the information is somewhat correct. | This is the correct answer, but the information is wrong. | It is the correct answer, and the information is accurate. |
| Usefulness | It has no educational pearls | There are about 1-2 educational pearls or important concepts that a competent physician should know. | There are quite a few (more than 3) educational pearls that a competent physician should know | The entire contents are educational pearls that a competent physician should know |
| Potential use in the classroom | No, I wouldn't use anything | I would use some of this as a guide | Yes, I would use the entire explanation |  |

Educational pearls are defined as must know concepts by medical doctors, for example “the most common cause of common cold is rhinovirus” is considered an educational pearl.
